# Is Crash Loading Acceptable in Carotid Artery Stenting? Results of Antiplatelet Crash Loading vs. Semi-crash vs. Elective Loading in a Large Study Population

**DOI:** 10.1007/s00062-022-01222-6

**Published:** 2022-10-20

**Authors:** Kamran Hajiyev, Hans Henkes, Viktoria Hellstern, Ali Khanafer, Christina Wendl, Hansjörg Bäzner, Philipp von Gottberg

**Affiliations:** 1 Neuroradiologische Klinik, Klinikum Stuttgart, Stuttgart, Germany; 2 Institut für Röntgendiagnostik, Zentrum für Neuroradiologie, Fakultät für Medizin, Universität Regensburg, Regensburg, Germany; 3 Neurologische Klinik, Klinikum Stuttgart, Stuttgart, Germany; 4 Medizinische Fakultät, Universität Duisburg-Essen, Essen, Germany

**Keywords:** Carotid artery stenting, Antiplatelet therapy, Stroke, Thrombocytic aggregation inhibition, Short-term loading, Double platelet inhibition

## Abstract

**Purpose:**

In elective carotid artery stenting (CAS), antiplatelet therapy (APT) is crucial. Several international societies have provided guidelines for loading time and dosage in endovascular treatment; however, no recommendations have been made for urgent, nonthrombectomy-associated CAS without adequate loading time. Here, we investigated the short-term outcomes for APT-naïve patients receiving “crash loading” (CL) on the day of intervention, compared with those for patients wi APT onset 3–5 days (semi-CL) or more than 5 days before CAS (EL).

**Methods:**

Outcomes of patients 30 days after CAS were evaluated in terms of the rates of in-stent thrombus, re-stenosis, stroke, hemorrhagic and thrombotic events, other periprocedural occurrences, in-hospital death and CAS-associated death. Patients’ biological, pathological and hemostatic factors were recorded and compared.

**Results:**

A total of 1158 patients who received CAS at the authors’ neuroradiology institution were analyzed: 275 EL, 846 semi-CL, and 37 CL. The patients receiving CL had the lowest rate of stroke, but the highest rates of CAS-associated and in-hospital deaths, although the deaths were not necessarily associated with APT. In-stent thrombosis was the highest in the semi-CL group. The rates and types of periprocedural occurrences favored the CL group.

**Conclusion:**

With the medical regimen used in this study, urgent CAS with CL APT did not produce more ischemic, thrombotic and hemorrhagic complications than longer loading times. However, careful patient selection might be crucial and adequate loading times should remain the standard of care.

## Introduction

In extracranial carotid artery stenting (CAS), inhibition of thrombocyte aggregation as antiplatelet therapy (APT) is the standard of care to prevent in-stent thrombosis and early in-stent stenosis. International societies for surgical and endovascular carotid artery treatment recommend dual APT pre- and posttreatment for internal carotid artery disease (ICAD), consisting of 81–325 mg aspirin and 75 mg clopidogrel per day, or 250 mg ticlopidine in the event of clopidogrel intolerance. According to the recommendations, dual APT should be started at least 3 days before the intervention and should be continued until at least 14 days afterward [1–4]. With the inclusion of this medical regimen as part of a multistep process before and after patient preparation, as well as attention to specific population and procedural aspects, like careful interdisciplinary patient selection, reducing the number of predilation maneuvers while avoiding postdilation, using closed cell stents etc. [5, 6], CAS has become a common treatment for ICAD, often performed in medium and large (neuro)interventional departments; it carries a low to very low risk of major infarction and death in elective, nonemergency settings [7–10].

In acute settings, CAS is often performed as part of thrombectomy in a so-called tandem occlusion, i.e., an occlusion of the proximal internal carotid artery followed by an occlusion of the ipsilateral terminal internal carotid artery and/or the middle cerebral artery. This maneuver is often described in the current literature and is usually considered feasible and safe [11–15]. However, CAS can also be performed in an acute setting without stroke, for example, in the context of urgent cardiac bypass surgery, bypass grafting or other major cardiovascular interventions with a risk of a prolonged decrease in blood pressure.

In patients with imminent bypass surgery, the role of carotid treatment and when to perform it is still controversial [16], but there seems to be a benefit of carotid treatment for patients with symptomatic stenosis prior to cardiac treatment [17–19]. To avoid the risk of stroke from cardiac treatment in the setting of concurrent ICAD, carotid artery screening and appropriate treatment are therefore routine procedures at many medical centers with a cardiovascular and neurointerventional focus.

However, despite the seemingly crucial use of CAS in such acute settings, little to no data are available on the efficacy and risks of medicines and dosages. Moreover, recommendations or guidelines are lacking regarding which medicines to use, at what dosage and when.

Therefore, in this study, we retrospectively analyzed data from APT-naïve patients who underwent urgent, nonthrombectomy-associated CAS with APT onset on the day of stenting. We compared the data with those for patients who qualified for elective CAS and had newly received APT 3–5 days before the intervention and for patients in an elective setting who had received APT for more than 5 days before CAS.

## Methods

### Patients

We analyzed data from all patients at our institution who received first time CAS treatment for ICAD between the years 2009 and 2020. We excluded patients receiving CAS as a primary treatment for acute cerebral ischemia or preparation of the way to treat acute ipsilateral cerebral ischemia, i.e. acute stroke treatment in the thrombectomy setting. Patients with symptomatic ICAD having a transient ischemic attack (TIA) until at least 7 days before or unchanged symptomatic since at least 7 days treatment were included in the study.

Patient medical history was analyzed with respect to body-mass index (BMI), diabetes, prior myocardial infarction and arterial hypertension. Patients’ sex and age were recorded, and the ICAD stenosis grade was classified according to the NASCET criteria [20]. We further recorded periprocedural data comprising the duration of intervention, applied radiation dosage, use of general vs. local anesthesia, use of prestenting balloon percutaneous transluminal angioplasty (PTA) vs. poststenting balloon PTA vs. prestenting and poststenting balloon PTA vs. no balloon PTA, the number of stents applied in the session and whether a protective device distal to the stenosis was applied.

Platelet inhibition was assessed on the day of intervention before CAS through VerifyNow™ (Werfen, Barcelona, Spain) and Multiplate® (Roche, Basel, Switzerland) tests whenever possible. Patients who had inadequate platelet inhibition in either test were excluded from CAS that day and further loaded, disqualifying them for inclusion in the APT-naïve, “crash loading” group.

Our institution is a tertiary referral center with an average of 1224 neurovascular interventions per year in the period of 2015–2021, including an average of 162 coiling procedures, 212 procedures with implantation of one or more flow-diversion units [21], an average of 376 thrombectomies and 212 primary carotid interventions per year. The catheter laboratory team consists of 6 interventionalists including 3 senior interventionalists.

### Medication

We divided the eligible patients into the following three groups:

1. Patients receiving APT more than 5 days before CAS (elective loading EL).
2. Patients receiving APT 3–5 days before CAS (semi-crash loading, SCL).
3. Patients receiving APT on the day of the CAS procedure, prior to the stent implantation (crash loading, CL).

Table 1 displays the administered drugs and dosages in the groups.

**Table 1:** Medical regimens in each group

Group	EL	Semi-CL	CL
APT drug #1	ASA 100 mg/d p.o.	ASA 100 mg/d p.o.	ASA 1 × 500 mg i.v.
APT drug #2	Clopidogrel 75 mg/d p.o. or ticagrelor 2 × 90 mg/d p.o. or prasugrel 10 mg/d p.o.	Clopidogrel 75 mg/d p.o. or ticagrelor 2 × 90 mg/d p.o. or prasugrel 10 mg/d p.o.	1/2009 to 5/2015: Clopidogrel 1 × 600 mg p.o. 6/2015 to 11/2020: ticagrelor 1 × 180 mg eptifibatide 1 × bolus 180 µg per kg bodyweight

*APT* antiplatelet therapy, *ASA* acetylsalicylic acid, *CL* crash loading, *EL* elective loading, *i.v*. intravenous, *p.o.* per os

During intervention and before stent implantation, fractionated heparin at a dose of 3000–5000 international units (IU) was administered whenever possible.

No recombinant tissue plasminogen activating factor was given.

In the medical regimen the day after treatment, acetylsalicylic acid (ASA) 100 mg/d p.o. and ticagrelor 2 × 90 mg/d or clopidogrel 75 mg/d were administered whenever possible.

### Endpoints and Follow-Up Assessment

The endpoints within the 30 day follow-up were as follows:

- Major hemorrhagic events, according to the definition from the International Society on Thrombosis and Hemostasis [22], with the exception of data collected 30 days after CAS instead of 5 half-lives after the latest administration of DAT before CAS.
- Any unusually prolonged puncture side bleeding, extensive groin hemorrhage and pseudoaneurysm formation detected by either sonographic imaging or computed tomography angiography.
- Any death associated with the CAS intervention.
- Any ipsilateral stroke resulting in permanent clinical deterioration.
- In-stent thrombosis.
- Any periprocedural complication leading to a major hemorrhagic event, major stroke or death.

### Statistics

Descriptive statistics were assessed as mean ± standard deviation for continuous variables and counts and percentages for categorical variables. Fisher’s exact test was used to determine the endpoint *p*-values; the threshold for statistical significance was set at 5% (α).

All statistical analyses were performed in R, version 4.0.2 (The R Foundation for Statistical Computing, Vienna, Austria).

## Results

**Fig. 1:**
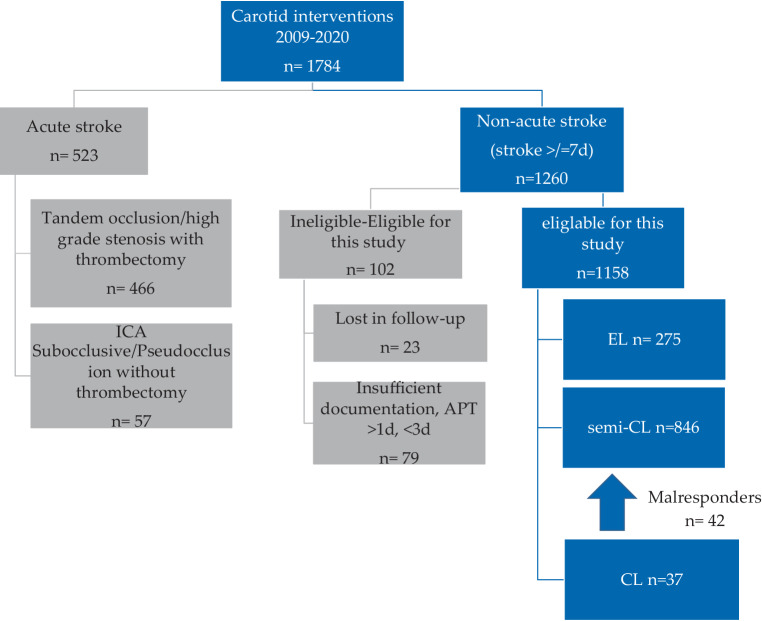
Cohort flow chart, *APT* antiplatelet therapy, *CL* crash loading, *d* day/days, *EL* elective loading, *ICA* internal carotid artery

A total of *n* = 1158 patients were eligible for inclusion in the study: *n* = 275 in the EL group, *n* = 846 in the semi-CL group and *n* = 37 in the CL group (Fig. 1). The data were analyzed in a retrospective, nonrandomized basis.

**Fig. 2:**
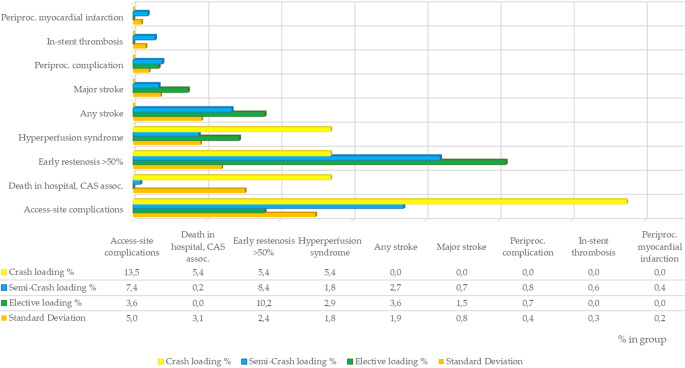
Key results in all three groups. *Assoc.* associated, *CAS* carotid artery stenting.

Fig. 2 displays the key results of the described groups.

ASA as first APT drug was administered from 2009 to 2020, clopidogrel as second APT drug was administered from 2009 to 2015 and replaced with ticagrelor or prasugrel as the standard in 2015 (see Table 1), resulting in less deferral of therapy due to clopidogrel low- or non-responders. Therefore, after 2015, more patients could be treated in a CL-status. Eptifibatide as a bolus in CL was applied throughout the whole evaluation period.

Until 2017, patient response to APT was tested at least on the day of intervention through multiple electrode aggregometry (Multiplate® test) whenever possible; from September 2017, the VerifyNow™ test was additionally performed whenever possible (s. Table 3); however, no change in peri- or postprocedural complications, patient recruitment, or other values observed in this study could be inferred from this, apart from the recording of additional data collected through the VerifyNow™ testing. The standard operation procedure for CAS was unaltered through the whole evaluation time and similar in all groups: General anesthesia was performed whenever possible. All cases considered for this investigation were treated by femoral approach, with a 7F-8F guiding catheter in the common carotid artery, balloon-catheter predilation of the stenosis if necessary and closed cell stents whenever possible. Consecutively, closed cell stent designs were dominant with a total of *n* = 961 (83% in total, *n* = 247/89.8% in EL, *n* = 679/80.3% in semi-CL and *n* = 35/94.6% in the CL group) with the Wallstent™ (Boston Scientific, Marlborough, USA) having the biggest share of 82.5% in total (*n* = 955, in EL *n* = 245/98.1%, in semi-CL *n* = 675/79.8% and in CL *n* = 35/94.6%). Open cell stent designs were used in a total of 17% (*n* = 197) with the biggest share in the semi-CL group (*n* = 167/19.7%, EL with *n* = 28/10.2%, CL with *n* = 2/5.4%).

Poststent dilation was to be avoided, protective devices were rarely used (*n* = 53/4.6% in total, *n* = 24/8.7% in EL, *n* = 29/3.4% in semi-CL and *n* = 0 in CL). There was no significant difference in outcome between the different stent types of each design. Due to the low number of times used, CAS procedures involving protective devices were not specifically evaluated.

CAS was performed by 6 different interventionalists during the evaluated period, including 3 main interventionalists responsible for over 70% of all cases. Because all interventions were overseen by at least one of the three lead interventionalists at our institution, results were not disaggregated by the interventionalist performing the intervention.

All patients were monitored in an intensive care unit for at least 24 h after the procedure, with continuous monitoring of vital signs and blood pressure limits not to exceed 130 mm Hg systolic. Patients underwent neurologic examination through an experienced board-certified neurologist within 12 h of CAS whenever possible and were frequently followed up by nurses. All patients underwent MRI or CT of the brain before discharge from the hospital.

The mean patient age at treatment was 71 years (range 41–96 years), and no significant difference was observed between groups. Most patients (70.5%; *n* = 816) were men: 69.5% in the EL group (*n* = 191), 70.7% in the semi-CL group (598) and 73% in the CL group (27). Arterial hypertension was the predominant concomitant disease, affecting at least 80% of each group and 82.6% in total (*n* = 957), whereas diabetes mellitus affected more than one third of patients in all groups (range 33.9–40.5%, *n* = 402). Myocardial infarction was present in 19.3% (range 18.1–24.3%, *n* = 223) of all patients, and the highest incidence was observed in the CL group.

The mean BMI was comparable in all groups (25 in the CL group vs. 26 in the EL and semi-CL groups). Only the maximum values were markedly higher in the EL and semi-CL groups than the CL group (43, 47 vs. 36, respectively).

Only 27 patients had tandem stenosis (mean 2.4%, range 1.5–2.7%), but 26.6% on average had a contralateral ICA occlusion (*n* = 136; mean 11.7%, range 10.6–24.3%; highest in the CL group) or stenosis above 50% according to NASCET criteria (*n* = 172; mean 14.9%, range 5.4–15.6%, lowest in the CL group). On average, 3.9% (*n* = 45) of the patients had a contralateral acute ischemic stroke preceding CAS. However, we did not distinguish between major and minor strokes, because none of the affected patients died or experienced an ipsilateral stroke during follow-up.

In 1.6% (*n* = 19) of patients, the grade of stenosis was below 50% according to NASCET criteria, and none of the patients received CL (EL *n* = 7, semi-CL *n* = 12). In 98.4% (*n* = 1139) of patients, the stenosis grade was above 50%; and among those patients, most had a stenosis grade of 70–99% (total 71.7%, *n* = 830). The number of patients with a stenosis grade of 70–99% was highest in the CL group (80.1%, *n* = 30) and was comparable between the EL and semi-CL groups (71.6%, 71.3%; *n* = 197, *n* = 603, respectively).

The mean treatment time was highest in the CL group, at 40 min (range 14–140 min), approximately 10 min more than the lowest mean treatment time among the three groups (EL group, mean 30 min, range 8–144 min). The periinterventional X‑ray exposure time was also highest in the CL group, with a mean of 22.3 min (range 4.7–69 min), followed by 19.4 min in the semi-CL (range 4–150 min) and EL (mean 18 min, range 3.9–109.1 min) groups. The balloon inflation pressure was comparable among all groups (mean 8 atm).

All patients showed sufficient platelet inhibition, as verified on the day of intervention before stent implantation with VerifyNow™ and Multiplate® tests, with a mean of 80–84% inhibition in the P2Y12 test (range 0.0–107%), and a median of 18–20 units (U) in the ADP test (range 0.0–142 U), and 12–14 U in the ASPI test.

No significant differences in platelet count were observed among the three groups, with a mean of 232 K/µL in the EL group, 236 K/µL in the semi-CL group and 227 K/µL in the CL group.

The mean hospital stay was shortest in the EL group, at 4 days (range 1–79 days), followed by the semi-CL group, at 5 days (range 0–50 days), and the CL group, at 7 days (range 1–49 days).

The percentages of patients with major stroke and stroke with any grade of severity were highest in the EL group, at 1.5%/3.6% (*n* = 4/10), followed by the semi-CL group, at 0.7%/2.7%, and the CL group, at 0%/0%. The CL group had the highest rate of CAS-associated in-hospital death, at 5.4% (*n* = 2), in comparison to 0.2% (*n* = 2) in the semi-CL group and 0% in the EL group. Both CAS-associated deaths in the CL group were due to severe cerebral hyperperfusion syndrome (CHS) immediately after the procedures, as were both deaths in the semi-CL group. Periprocedural complications occurred in a total of nine patients (0.8%), primarily in the semi-CL group (*n* = 7/0.8% vs. *n* = 9/0.8% in EL and *n* = 0/0% in CL, respectively). None of the complications resulted in major stroke or death. Clinical symptoms and/or MRI signs of CHS occurred in 25 of patients (2.2%), primarily in the CL group, at *n* = 2 (5.4%), followed by the EL group, at *n* = 8 (2.9%), and the semi-CL group, at *n* = 15 (1.8%). As described above, four patients died because of this condition on the day of, or few days after, the intervention.

Acute periprocedural myocardial infarction was observed exclusively in the semi-CL group, with a total of *n* = 3 (0.4% in the group, 0.3% in the total study population). All three patients suffered from chronic coronary artery disease.

Access site complications following the intervention, such as prolonged bleeding, the dissection of the access vessel, puncture site thrombosis or pseudoaneurysm formation, were most frequently reported in the CL group (*n* = 5, 13.5%) and semi-CL group (*n* = 63, 7.4%), but were comparably rare in the EL group (*n* = 10, 3.6%). In the CL group, the types of access site complications were pseudoaneurysm in 5% (*n* = 2) and groin hematoma in 8% (*n* = 3). All five patients were treated conservatively, and none required surgery.

Residual stenosis of ≥ 50% was avoided in nearly all cases and was seen most frequently in the semi-CL group (*n* = 4, 0.5%) but did not occur in the CL group. However, early restenosis, as defined by 50% or more reduction of the vessel diameter following the NASCET criteria on MRI/CT before discharge, was most frequently seen in patients receiving elective procedures (10.2%), followed by those receiving semi-CL (8.4%); the lowest rate was observed in the patients receiving CL (5.4%). In-stent thrombosis occurred most frequently in the semi-CL group, at *n* = 5 (5.4%), but was rare overall (*n* = 14, 1.2%).

Tables 2, 3, 4, 5 and 6 display all the results.

**Table 2:** Biological factors, comorbidities, and medications

		Total (*n* = 1158)	Elective (*n* = 275)	Semi-crash (*n* = 846)	Crash (*n* = 37)
Sex	Female	342 (29.5%)	84 (30.5%)	248 (29.3%)	10 (27.0%)
	Male	816 (70.5%)	191 (69.5%)	598 (70.7%)	27 (73.0%)
DM	Yes	402 (34.7%)	100 (36.4%)	287 (33.9%)	15 (40.5%)
Arterial hypertension	Yes	957 (82.6%)	231 (84.0%)	696 (82.3%)	30 (81.1%)
CAD	Yes	450 (38.9%)	110 (40.0%)	324 (38.3%)	16 (43.2%)
Myocardial infarction	None	935 (80.7%)	214 (77.8%)	693 (81.9%)	28 (75.7%)
	Yes	223 (19.3%)	61 (22.2%)	153 (18.1%)	9 (24.3%)
Periprocedural heparin	No	11 (0.9%)	1 (0.4%)	6 (0.7%)	4 (10.8%)
	Heparin 3000 IE	415 (35.8%)	179 (65.1%)	223 (26.4%)	13 (35.1%)
	Heparin 5000 IE	732 (63.2%)	95 (34.5%)	617 (72.9%)	20 (54.1%)
Grade of Stenosis following NASCET criteria	< 50%	19 (1.6%)	7 (2.5%)	12 (1.4%)	0 (0%)
	50–69%	309 (26.7%)	71 (25.8%)	231 (27.3%)	7 (18.9%)
	70–99%	830 (71.7%)	197 (71.6%)	603 (71.3%)	30 (81.1%)
Contralat. ICA-Occl	No	1022 (88.3%)	246 (89.5%)	748 (88.4%)	28 (75.7%)
	Yes	136 (11.7%)	29 (10.6%)	98 (11.6%)	9 (24.3%)
Contralateral ICA-Stenosis	No	805 (69.5%)	174 (63.3%)	598 (70.7%)	33 (89.2%)
	50–75%	126 (10.9%)	28 (10.2%)	98 (11.6%)	0 (0%)
	> 75%	46 (4.0%)	10 (3.6%)	34 (4.0%)	2 (5.4%)
Tandem stenosis	Yes	17 (2.4%)	4 (1.5%)	22 (2.6%)	1 (2.7%)

*CAD* coronary artery disease, *DM* diabetes mellitus, *ICA* internal carotid artery, *NASCET* north american symptomatic carotid endarterectomy trial

**Table 3:** Median and minimum-maximum values of biological, pathological and hemostatic factors

	Loading	*n*	Mv	SD	Median	Min-Max
Age at treatment	Elective	275	71.2	9.0	72.0	46.0–90.0
	Semi-crash	846	70.2	9.6	71.0	41.0–96.0
	Crash	37	71.6	10.5	71.0	45.0–87.0
BMI	Elective	275	26.5	4.2	26.0	15.0–43.0
	Semi-crash	846	26.7	3.6	26.0	16.0–47.0
	Crash	37	25.9	3.7	25.0	21.0–36.0
Platelet count (K/µL)	Elective	275	244.0	67.1	232.0	41.0–553.0
	Semi-crash	846	253.0	84.9	236.0	63.0–1173.0
	Crash	37	240.2	78.0	227.0	119.0–505.0
ADP test (U)	Elective	261	24.1	18.7	19.0	0.0–142.0
	Semi-crash	413	24.6	18.9	20.0	0.0–124.0
	Crash	19	22.7	18.7	18.0	2.0–80.0
ASPI test (U)	Elective	261	17.4	28.5	13.0	0.0–20.0
	Semi-crash	412	16.2	16.8	12.0	0.0–119.0
	Crash	18	13.2	9.6	14.0	0.0–29.0
ARU test	Elective	154	439.8	75.8	399.5	350.0–629.0
	Semi-crash	173	449.8	79.7	416.0	283.0–620.0
	Crash	9	436.3	88.4	392.0	348.0–584.0
P2Y12 inhibition (%)	Elective	161	81.9	18.2	84.0	0.0–99.0
	Semi-crash	180	78.7	20.5	80.0	0.0–107.0
	Crash	9	76.3	24.8	84.0	26.0–97.0
NASCET (%)	Elective	275	75.0	14.4	75.0	20.0–99.0
	Semi-crash	846	75.2	13.6	75.0	30.0–99.0
	Crash	37	79.1	12.7	80.0	50.0–97.0
Hospital stay	Elective	275	6.9	7.2	4.0	1.0–79.0
	Semi-crash	846	7.5	6.2	5.0	0.0–50.0
	Crash	37	9.9	9.3	7.0	1.0–49.0
Treatment time	Elective	275	33.0	17.2	30.0	8.0–144.0
(minutes)	Semi-crash	846	37.8	18.9	35.0	8.0–180.0
	Crash	37	45.4	25.9	40.0	14.0–140.0
X‑ray exposure time	Elective	271	20.5	13.6	18.0	3.9–109.1
(minutes)	Semi-crash	503	21.7	13.7	19.4	4.0–150.0
	Crash	35	27.2	15.9	22.3	4.7–69.0

*ADP* adenosine diphosphate, *ARU* aspirin reactivity units, *ASPI* arachidonic acid stimulated platelet inhibition, *BMI* body-mass index, *Mv* mean value, *NASCET* north american symptomatic carotid endarterectomy trial, *P2Y12* chemoreceptor for adenosine diphosphate, *SD* standard deviation

**Table 4:** Indications for carotid artery stenting in patients with internal carotid artery stenosis (imminent cardiovascular surgery in parentheses)

	*n* total	*n* EL	*n* Semi-CL	*n* CL
Ipsil. stroke >/= 7d	214	39	155	20 (7)
Contralat. hemodynamic stroke at contralat. ICA occlusion/stenosis	145	42	103	0
TIA	118	16	97	5 (2)
Amaurosis fugax	45	8	36	1
Asymptom. > 75% stenosis	636	170	344	11 (9)

*d* day/days, *ICA* internal carotid artery, *TIA* transient ischemic attack

**Table 5:** Endpoints

	Total (*n* = 1158)	Elective (*n* = 275)	Semi-crash (*n* = 846)	Crash (*n* = 37)	*p*-value*
Major stroke	10 (0.9%)	4 (1.5%)	6 (0.7%)	0 (0%)	0.474
In-hospital death	9 (0.8%)	0 (0%)	7 (0.8%)	2 (5.4%)	0.010
Major stroke, in-hospital death	19 (1.6%)	4 (1.5%)	13 (1.5%)	2 (5.4%)	0.183
Major stroke, CAS-associated death	14 (1.2%)	4 (1.5%)	8 (0.9%)	2 (5.4%)	0.059
Major stroke, minor stroke	33 (2.8%)	10 (3.6%)	23 (2.7%)	0 (0%)	0.538
Minor/major stroke, in-hospital death	42 (3.6%)	10 (3.6%)	30 (3.5%)	2 (5.4%)	0.732

* *p*-value of Fisher’s exact test

**Table 6:** Secondary endpoints

	Total (*n* = 1158)	Elective (*n* = 275)	Semi-crash (*n* = 846)	Crash (*n* = 37)	*p*-value*
Periprocedural complication	9 (0.8%)	2 (0.7%)	7 (0.8%)	0 (0%)	1.000
Hyperperfusion syndrome	25 (2.2%)	8 (2.9%)	15 (1.8%)	2 (5.4%)	0.149
Access site complications	78 (6.7%)	10 (3.6%)	63 (7.4%)	5 (13.5%)	0.015
Acute periprocedural myocardial infarction	3 (0.3%)	0 (0%)	3 (0.4%)	0 (0%)	1.000
Restenosis	101 (8.7%)	28 (10.2%)	71 (8.4%)	2 (5.4%)	0.585
In-stent thrombosis	5 (0.4%)	0 (0%)	5 (0.6%)	0 (0%)	0.086
Residual stenosis ≥ 50%	5 (0.4%)	1 (0.4%)	4 (0.5%)	0 (0%)	1.000

* *p*-value of Fisher’s exact test

## Discussion

In terms of age and comorbidities, patients were largely comparable across the EL, semi-CL and CL groups. The mean age, the higher prevalence of men, the large proportion of patients with arterial hypertension and the prevalence of diabetes in our study population were also comparable, and have been the main reported comorbidities in patient cohorts in major trials investigating elective CAS [10, 23–25].

A small rate of early in-stent thrombosis was observed, which were found exclusively in the semi-CL group. This focus on the semi-CL group could not be explained by potentially poorly adjusted APT or patients being low responders, because the VerifyNow™ and Multiplate® tests ruled out patients with insufficient platelet inhibition and indicated a balanced level of APT among all groups on the day of intervention. In addition, the stent types and CAS techniques did not differ between the groups, and the finding can most likely be explained by the misbalance in patient numbers included in the groups with the highest number in the semi-CL group.

It is also remarkable that no such thrombotic event was observed in the CL group, which was different from the EL and semi-CL group in the way that an urgent intervention of a non-neurovascular type, like major cardiovascular procedures, was performed shortly after CAS in several patients. Thus, patients in the CL group might have had higher inflammatory factors and hypercoagulability in the short clinical course, a phenomenon well described and discussed in the literature [26–30], raising the risk for early in-stent thrombosis.

When considering early restenosis of carotid stents as a result of extensive thrombocyte aggregation on foreign surface and a preliminary stage of thrombosis, in the context of the finding described above, the comparatively low restenosis rate in the CL group, also despite having the highest average grade of stenosis, was remarkably and does not reflect the putative higher tendency of CL patients to accumulate thrombocytic plaque on the stent surface because of a supposedly insufficient APT exposure time. The exclusion of patients with insufficient platelet inhibition through testing prevented CAS in possible low and no responders, however, and may therefore have had a major impact on the low rates of thrombotic complications in the CL group.

Unusually prolonged bleeding and groin hematoma as access site complications after the intervention were reported frequently in the CL groupowever, other than in the EL and semi-CL group, all patients were treated successfully with conservative means, and none of the patients in the CL group had major hemorrhage, as defined by the International Society on Thrombosis and Hemostasis. The access site complication rate at our institution (total of 6.7%) is slightly above that reported in the literature [31, 32], which also varies between studies, possibly because of varying levels of training among interventionalists. However, the staff at our institution, being a high-load center, could be considered to have above-average experience, as reflected by the lower than average periprocedural complications and deaths, as described above. Still, a possible trade off may be the slightly higher number of access site complications.

The rates of in-hospital and CAS-associated deaths were also remarkably high in the CL group, as was the percentage of patients with CHS. However, these observations are biased in our data, because both of the patients in the CL group who had CHS died as a result. CHS is a well described yet not fully understood phenomenon that can appear after CAS and carotid thrombendarterectomy, and is thought to be caused mainly by poor blood pressure management [33–35]. The data in this study again indicate the absolute necessity for preparation and careful supervision of patients early after carotid artery intervention to prevent CHS.

No other cases of in-hospital death and CAS-associated death occurred in the CL group, and the rates of major stroke and all stroke were also favorable in the CL group. However, these findings might have been due to the limited number of cases in the CL group. In the EL and semi-CL group, which had relatively high case numbers, the rates of major stroke, any stroke, in-hospital death and CAS associated death at our institution remain below those in major trials, such as those described earlier in this section. The number of patients receiving CL was too few to allow a valid estimation; however, on the basis of the current results, we estimated that the rate of stroke and/or death in the CL group might have been equal to, rather than much higher than, those in other two groups.

Summing up the general limitations of this study, the misbalance of case numbers in the observed groups is striking. In particular, the preponderance of semi-CL case series may confound the results and interpretation of this study. Also, the study design is retrospective and there has not been any kind of patient randomization. Patients were selected consecutively based on an interdisciplinary case by case decision, resulting in reduced homogeneity of compared items. The key group of CL patients is therefore comparably small, but to the best of the authors’ knowledge, no trial, cohort study, or equivalent investigation has yet addressed this problem, which most neurointerventionalists frequently face, in this manner. Comparatively constant periprocedural management and CAS procedures by a limited number of interventionalists over time at a single center generates limited comparability and enables a trend assessment.

However, the data is yet not reliable enough for a definite assessment of the impact of loading time on the outcome of CAS.

## Conclusion

In elective CAS, an adequate loading time for APT, consisting of several days, is considered the standard of care. However, in cases in which carotid artery rehabilitation is urgently needed, the medical regimen and careful selection of patients described in this study did not result in higher rates of severe strokes, minor strokes, thrombotic and hemorrhagic events.

On the basis of the assumption that CHS after CAS is caused mainly by uncontrolled hypertension and is less affected by the APT loading time, also the risk of in-hospital and CAS-associated death might not actually be higher with “crash-loading” than with longer loading times of APT.

However, because the number of patients in the different groups in this study is imbalanced, the data are not yet strong enough for a definitive evaluation of the safety of “crash loading” APT in CAS, and elective loading should remain the standard of care.
